# Retrospective Comparative Analysis of Clinical and Imaging Features of Craniocervical Artery Dissection: Spontaneous CAD vs. Minor Traumatic CAD

**DOI:** 10.3389/fneur.2022.836997

**Published:** 2022-03-16

**Authors:** Dan Xu, Yongjun Wu, Jingjing Li, Shihui Xing, Hongbing Chen, Xinran Chen, Yan Tan, Kun Zhou, Guofen Zhang, Jian Zhang

**Affiliations:** ^1^Department of Neurology, The First Affiliated Hospital, Sun Yat-sen University, Guangdong Provincial Key Laboratory for Diagnosis and Treatment of Major Neurological Diseases, National Key Clinical Department and Key Discipline of Neurology, Guangzhou, China; ^2^Curtin Medical School/Curtin School of Population Health, Faculty of Health Sciences, Curtin University, Perth, WA, Australia; ^3^Medical Education and General Practice, The First Affiliated Hospital, Sun Yat-sen University, Guangzhou, China; ^4^Department of Neurology, First Affiliated Hospital of Guangxi University of Chinese Medicine, Nanning, China

**Keywords:** craniocervical arterial dissection, ischaemic stroke, cervical manipulation, minor trauma dissection, spontaneous dissections

## Abstract

**Background and Objectives:**

Craniocervical artery dissection (CAD) is the most common cause of ischemic stroke in young adults. The etiologies of CAD can be classified into three types, such as spontaneous (sCAD), minor traumatic (mtCAD), and genetic origin. Recent studies indicated that clinical presentations and imaging features could guide management and inform prognosis. This retrospective analysis sought to compare the clinical and imaging features of sCAD vs. mtCAD in providing evidence-based advice on medical treatment, functional rehabilitation, secondary stroke prevention, and prognosis, ultimately formulating clinical guidelines in managing CAD.

**Methods:**

In total, 148 patients with CAD were identified from the medical records database and subdivided into sCAD and mtCAD based on the clinical presentations and imaging features. A retrospective comparative analysis was performed according to their clinical presentations and imaging features.

**Results:**

Patients with mtCAD are significantly younger than sCAD with 120 cases of sCAD average aged 43.61 ± 12.75, while 28 cases of mtCAD average aged 35.68 ± 14.54. Patients with mtCAD had more cases of neck pain compared to sCAD. Patients with mtCAD had more cases of CAD at extracranial locations compared to sCAD. Patients with mtCAD had more cases of multiple site dissection compared to sCAD. Double lumen and intramural haematoma are the most common imaging findings with mtCAD patients having statistical significantly more cases of intramural haematoma and long tapering stenosis.

**Conclusion:**

Patients with mtCAD were presented at a much younger age with symptoms of neck pain compared to sCAD. Patients with mtCAD predominantly presented at extracranial sites with more prominent features of multiple site dissection, intramural haematoma, and long tapering stenosis. These clinical and imaging features can translate into clinical practice guidelines for patients with CAD to improve the optimal functional outcome and reduce both morbidity and mortality.

## Introduction

Craniocervical artery dissection (CAD) is characterized by the tearing of either the intimal, medial, or adventitial layers of the walls of the internal carotid or vertebral arteries, accounting for up to 10–25% of ischaemic stroke in young- to middle-aged individuals under aged 45 during the fourth and fifth decades (1). CAD in the traditional literature is often considered to present as spontaneously type with an annual incidence of 2.5–3:10,000 (2).

Craniocervical artery dissection is the second most common large-artery cerebrovascular disease after atherosclerosis with average onset age of 51 in vertebral artery dissection and 43 in carotid dissections. CAD is considered to be either spontaneous or traumatic or genetic in origin. Although a significant number of spontaneous dissections are idiopathic or actually genetic, underlying pathologies possibly, such as hypertension, atherosclerosis, fibromuscular dysplasia, type IV Ehlers-Danlos syndrome, Marfan syndrome, type I osteogenesis imperfecta, alfa-1-antitrypsin deficiency, cystic medial necrosis, autosomal dominant polycystic renal disease, medial mucoid degeneration, polyarteritis nodosa, Behçet's disease, migraine, transient postinfectious arteriopathy, and long styloid process, may be identified as the causes or associated risk factors (3, 4). A recent research suggested that infection could precipitate dissection, probably associated with seasonal variation of spontaneous CAD (sCAD), demonstrating a peak in autumn and winter (5). sCAD has also been reported to be preceded by trivial trauma, such as the possibilities of coughing, vomiting, sneezing, fast head turning, neck massage, in a susceptible individual with an underlying arteriopathy (1, 6, 7). The more recent study of Cervical Artery Dissection and Ischemic Stroke Patients (CADISP) analyzed 982 patients with cervical artery dissection in terms of trauma severity. Severe trauma accounts for 4.5% of carotid artery dissection and 5.6% of vertebral artery dissection, while minor trauma accounts for 29.2% of carotid artery dissection and 36.5% of vertebral artery dissection (8). A statement from American Stroke Association indicated the need for risk discussion of minor traumatic CAD (mtCAD) by cervical massage and manipulation (7). It is clear that mtCAD presented more commonly with difficulty in differentiating from sCAD because of limited understanding of mtCAD risk factors, clinical and imaging features to guide management. Our study is aimed to compare and analyse the clinical and imaging features of sCAD vs. mtCAD with the subsequent formulation of the evidence-based guideline on medical treatment, functional rehabilitation, secondary stroke prevention, and prognosis advice.

## Methods

### Participants

**Figure 1:**
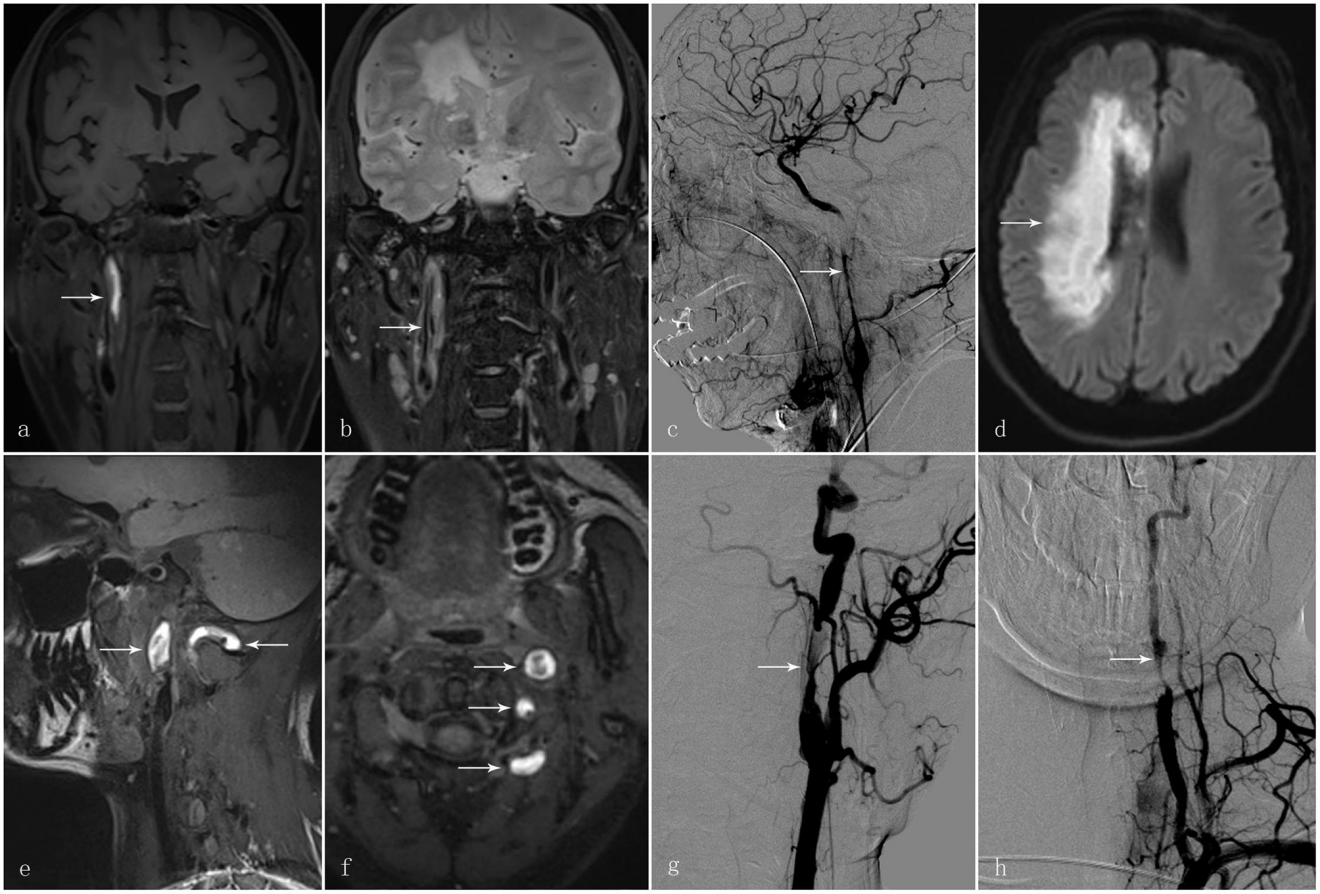
A 58-year-old man presented with sudden-onset left hemiparesis and paraesthesia. **(a)** Coronal view of the high-resolution MRI T1 SPACE images showed an intramural hematoma at the C1 segment of the right internal carotid artery [**(a)**; arrow]. **(b)** T2 images demonstrated intimal flap and double lumen in the corresponding vessel wall [**(b)**; arrow]. **(c)** Digital subtraction angiography in the same patient showed long tapering stenosis at the C1 segment of the right internal carotid artery [**(c)**; arrow]; **(d)** Diffusion-weighted imaging demonstrated an acute infarct in the territory of the right internal carotid artery [**(d)**; arrow]. Another 30-year-old man presented with vertigo and vomiting after cervical manipulation. **(e, f)** The high-resolution MRI T1 SPACE images showed the intramural hematoma at the left internal carotid artery and vertebral artery [**(e, f)**; arrows]. **(g)** Digital subtraction angiography displayed irregular long tapering stenosis at the C1 segment of the left internal carotid artery and V2 segment of the left vertebral artery **[(g, h)**; arrow].

This retrospective study was approved by the Institutional Review Board of The First Affiliated Hospital of Sun Yat-sen University with written informed consent regarding data use for research purposes being obtained from each of 148 participants. In total, 148 participants diagnosed with CAD were selected *via* the medical record system of The First Affiliated Hospital of Sun Yat-sen University, between January 2010 and January 2020 with the diagnosis of CAD being confirmed by two stroke neurologists and a neuro-radiologist. The diagnostic confirmation of CAD was defined by the following criteria: intramural hematoma, intimal flap, double lumen, long tapering stenosis, artery occlusion that recanalised in an irregular aneurysmal dilation or stenosis, or irregular aneurysmal dilation with associated filiform and irregular stenosis on MRI and angiography (MRA), or CT angiography (CTA), or digital subtraction angiography (DSA) (1, 9). Two typical cases are illustrated in Figure 1.

### Inclusions and Exclusions

Patients were included as eligible participants by their clinical and imaging features of CAD based on the above-mentioned diagnostic criteria. Patients were excluded if they had other occlusive vascular diseases such as atherosclerosis and vasculitis, or if they had CAD from severe trauma and iatrogenic causes, or if medical records were incomplete.

### Data Collection

All participants during their hospitalization were provided with a set of evidence-based standardized questionnaires recording activities of abrupt cervical movements, cervical manipulation, sporting activities involving head and neck movement, impact injury of the head and neck within 1 month before the onset of artery dissection (10).

All participants' demographic data and lifestyle risk factors were extracted from the medical records, such as age, sex, hypertension, diabetes, alcohol drinking, smoking, cervical massage, and head or cervical trauma. Total cholesterol, triglycerides, low-density lipoprotein cholesterol (LDL-C), high-density lipoprotein cholesterol (HDL-C), and blood glucose levels were also collected. Hypertension was defined as a history of systolic blood pressure ≥140 mmHg and/or diastolic blood pressure ≥90 mmHg, or if the patient was taking antihypertensive drugs. Diabetes was defined by a history of fasting blood glucose ≥7.0 mmol/L, 2 h postprandial ≥11.1 mmol/L, or the use of hypoglycaemic therapy. All data were measured within 48 h of hospitalization. We defined current smokers as individuals who smoked any tobacco in the past 12 months and included those who had quit within the past year. Alcohol consumption was recorded as past or present drinking (more than 1 drink per month) (11). The definition of mtCAD is based on trauma by direct or indirect impact to the head and neck and any mechanism spuriously raising intra-thoracic pressure, such as whiplash, violent coughing, abrupt neck movement, and head and neck manipulation within 1 month prior to the CAD (6, 7). After reviewing the questionnaires in the medical record, two authors discussed and decided whether the patients were mtCAD or sCAD. The severity of stroke associated with any functional impairment at admission was evaluated using the National Institutes of Health Stroke Scale (NIHSS) and modified Rankin Scale Score (MRS), respectively. The NIHSS and MRS were reassessed on discharge with follow-up at 6 and 12 months after the onset of symptoms.

### Statistical Analysis

The demographic data, clinical and imaging features were analyzed between the sCAD and mtCAD groups. Quantitative data of normal distribution were expressed as mean ± SD and analyzed using Student's *t-*test. Quantitative data of skewed distribution were presented as median and quartile and analyzed using the Wilcoxon rank-sum test. Qualitative data were expressed as frequency and percentage (%), and the chi-square test or Fisher's exact test was used for statistical difference analysis. All data were analyzed using GraphPad Prism Version 8.0 software (Graph Pad Software Inc., San Diego, CA, USA). A *p* of < 0.05 was considered statistically significant.

## Results

### Demographic Data and Clinical Features

In total, 148 patients (Table 1) were included with 120 patients with sCAD (95 men and 25 women, average aged 43.61 ± 12.75, median age 44.50) and 28 patients with mtCAD (20 men and 8 women, average aged 35.68 ± 14.54, median age 35.50) accounting for 18.9% of all patients with CAD. In terms of the age of onset with CAD, patients with mtCAD were significantly younger than sCAD patients (*p* = 0.0045). In total, 28.57% of the patients with mtCAD were presented with neck pain, compared to 5.83% of the patients with sCAD (*p* = 0.0003). There were no statistically significant differences between mtCAD and sCAD patients in terms of gender and the associated risk factors of hypertension, diabetes, smoking, alcohol consumption, headache, total cholesterol, triglycerides, LDL-C, HDL-C, and C-reactive protein (Table 1).

**Table 1:** Demographic data and clinical features.

	**mtCAD (*n* = 28)**	**sCAD (*n* = 120)**	**OR (95%CI)**	***P*-value**
Age (years), mean ± SD (Median)	35.68 ± 14.54 (35.50)	43.61 ± 12.75 (44.50)	1.05 (1.01–1.08)	0.0045
Male (%)	20 (71.43%)	95 (79.17%)	0.66 (0.26–1.65)	0.3757
Hypertension (%)	4 (14.29%)	30 (25.00%)	0.50 (0.18–1.44)	0.2249
Diabetes (%)	0 (0%)	6 (5.00%)	0.00 (0.00–3.15)	0.2271
Smoking (%)	6 (21.43%)	48 (40.00%)	0.41 (0.16–1.05)	0.0660
Drinking (%)	6 (21.43%)	36 (30.00%)	0.64 (0.24–1.67)	0.3650
Headache (%)	10 (35.71%)	50 (41.67%)	0.78 (0.33–1.75)	0.5635
Cervical pain (%)	8 (28.57%)	7 (5.83%)	6.46 (2.03–18.37)	0.0003
Horner syndrome (%)	2 (7.14%)	11 (9.17%)	0.76 (0.16–3.13)	0.7334
Total cholesterol (mmol/L)	3.74 ± 0.98	4.20 ± 1.24	1.32 (1.01–1.72)	0.0858
Triglycerides (mmol/L)	1.39 ± 0.93	1.38 ± 0.78	1.12 (0.67–1.88)	0.9847
HDL-C (mmol/L)	1.02 ± 0.24	1.07 ± 0.24	2.37 (0.77–7.30)	0.3016
LDL-C (mmol/L)	2.22 ± 0.61	2.60 ± 0.99	1.56 (1.03–2.37)	0.0678
CRP (mmol/L)	3.39 ± 7.07	6.11 ± 11.52	1.04 (0.97–1.10)	0.2895

*HDL-C, high-density lipoprotein cholesterol; LDL-C, low-density lipoprotein cholesterol; CRP C, reactive protein*.

### Imaging Features

Among the 148 patients with CAD, 75 patients had an internal carotid artery dissection and 54 patients had a vertebral artery dissection. No statistical significance was observed between mtCAD and sCAD patients in terms of the frequencies of dissections in the internal carotid artery, vertebral artery, middle cerebral artery, and basilar artery (Table 2). Among all patients with CAD, 91 patients had extra-cranial CAD with 57 patients with intracranial CAD. Of the mtCAD patients, 22 patients (78.57%) had extra-cranial dissection and 6 patients (21.43%) with intracranial dissections, while 69 sCAD patients (57.5%) had extra-cranial dissections and 51 sCAD patients (42.5%) with intracranial dissections. In statistical terms, patients with mtCAD had significantly more extra-cranial dissections in contrast with patients with sCAD having significantly more intracranial dissections, representing a statistically significant difference (*p* = 0.0391). In terms of CAD in multiple arteries, 19 patients had multiple CADs, such as 7 patients with mtCAD (25%) having significantly more dissections compared to patients with sCAD (*p* = 0.0326). The two most common imaging features of CAD include “double lumen” (31.75%) and intramural haematoma (26.35%). The two most striking imaging features of dissections were “string sign” and “intramural haematoma” in the 148 patients with CAD. In terms of the imaging feature of “string sign,” mtCAD appeared in 10 patients (35.71%) and was statistically different compared to 19 sCAD patients (15.83%) with “string sign” (*p* = 0.017). With respect to the imaging feature of “intramural haematoma,” 12 patients with mtCAD (42.86%) had imaging feature of “intramural haematoma” in contrast to 27 patients with sCAD (22.50%) with imaging feature of “intracranial haematoma,” reaching statistical significance (*p* = 0.0277). However, imaging features of “double lumen,” “lumen irregular stenosis,” “dissecting aneurysms,” and “intimal flap” did not show a statistical difference between mtCAD and sCAD patients (Table 2).

**Table 2:** Imaging features.

	**Total (*n* = 148)**	**mtCAD (*n* = 28)**	**sCAD (*n* = 120)**	**OR (95%CI)**	***P*-value**
Carotid artery dissection	75 (50.67%)	16 (57.14%)	59 (49.17%)	1.38 (0.62–3.16)	0.4472
Vertebral artery dissection	54 (36.49%)	14 (50.00%)	40 (33.33%)	2.00 (0.87–4.61)	0.0990
MCA dissection	17 (11.41%)	3 (10.34%)	14 (11.67%)	0.87 (0.25–2.97)	0.8408
Basilar artery dissection	7 (4.73%)	0 (0%)	7 (5.83%)	0 (0–2.43)	0.1904
Left-sided	81 (54.73%)	20 (71.43%)	61 (50.83%)	2.42 (1.03–5.71)	0.0487
Right-sided	74 (46.84%)	13 (34.21%)	61 (50.83%)	0.50 (0.24–1.10)	0.0735
Multiple dissection	19 (12.84%)	7 (25.00%)	12 (10.00%)	3.00 (1.14–8.05)	0.0326
Intracranial dissection	57 (41.22%)	6 (21.43%)	51 (42.50%)	0.37 (0.14–0.95)	0.0391
Extracranial dissection	91 (58.78%)	22 (78.57%)	69 (57.50%)	2.71 (1.06–6.96)	0.0391
Intracranial extension	21 (14.19%)	3 (10.715%)	18 (15.00%)	0.68 (0.20–2.48)	0.5584
Cerebral infarction	126 (85.13%)	27 (96.43%)	99 (82.50%)	5.7 (0.91–61.60)	0.0621
TIA	5 (3.38%)	0 (0%)	5 (4.17%)	0.00 (0.00–2.97)	0.2718
SAH	4 (2.71%)	1 (3.57%)	3 (2.50%)	1.44 (0.11–9.98)	0.7529
Double lumen	47 (31.75%)	6 (21.43%)	41 (34.17%)	0.53 (0.20–1.37)	0.1923
Irregular stenosis	35 (23.63%)	6 (21.43%)	29 (24.17%)	0.86 (0.32–2.32)	0.7588
String sign	29 (19.60%)	10 (35.71%)	19 (15.83%)	2.95 (1.23–6.97)	0.0170
Dissecting aneurysms	28 (18.92%)	3 (10.71%)	25 (20.83%)	0.46 (0.14–1.55)	0.2183
Intimal flap	13 (8.79%)	5 (17.86%)	8 (6.67%)	3.04 (1.01–9.02)	0.0596
Intramural hematoma	39 (26.35%)	12 (42.86%)	27 (22.50%)	2.58 (1.14–6.15)	0.0277

*MCA, middle cerebral artery; TIA, transient ischemic attack; SAH, subarachnoid hemorrhage*.

### Intervention

Among the 6 patients who underwent thrombolysis treatment, there were 5 sCAD and 1 mtCAD patients. In total, 15 sCAD and 2 mtCAD patients had vascular angioplasty procedures as interventions. Both interventions did not demonstrate any statistical significance of functional improvements with evaluation by NIHSS at discharge in both sCAD and mtCAD patients (Table 3). In addition, 74.17% of sCAD and 67.86% of mtCAD patients had a favorable outcome when MRS is less or equal to 2 (MRS ≤ 2) at admission. During the follow-up, 17 sCAD and 4 mtCAD patients were lost due to the change of telephone number. Nevertheless, the proportion of patients with a favorable functional outcome was reached 82.52 and 91.67% in sCAD and mtCAD at 12 months, respectively. No significant difference was found in terms of functional outcome in sCAD and mtCAD patients by applying different interventions (Table 3).

**Table 3:** Intervention and outcomes.

	**mtCAD**	**sCAD**	**OR**	***P*-value**
	**(*n* = 28)**	**(*n* = 120)**	**(95%CI)**	
Intravenous thrombolysis	1(3.57%)	5(4.17%)	0.85(0.07–6.77)	0.8857
Endovascular procedures	2(7.14%)	15(12.50%)	0.54(0.12–2.27)	0.4234
Initial NIHSS	6.43 ± 6.20	5.68 ± 6.00	0.98(0.92–1.05)	0.5575
NIHSS at discharge	4.32 ± 4.97	3.67 ± 4.53	0.97(0.89–1.06)	0.4998
Initial MRS (≤ 2)	19(67.86%)	89(74.17%)	0.74(0.31–1.74)	0.4984
6-month MRS (≤ 2)	21(87.50%)	78(75.73%)	2.25(0.63–7.58)	0.2103
12-month MRS (≤ 2)	22(91.67%)	85(82.52%)	2.33(0.56–10.72)	0.2681

*NIHSS, National Institutes of Health Stroke scale; MRS, modified Rankin Scale*.

## Discussion

With the technological advancement of modern imaging, CAD has been increasingly recognized as a major cause of ischemic stroke in young adults (12, 13). In this study, the mean age of 148 patients was below 50 years with patients with sCAD and mtCAD averaged age being 43 and 35 years, respectively, which were consistent with previous reports (8, 10). Patients with mtCAD were significantly younger than patients with sCAD, pointing to the fact of increased prevalence of minor trauma associated with the more active lifestyle in younger patients (10). The Cervical Artery Dissection in Ischaemic Stroke Patients (CADSIP) study and an earlier study indicated that most CAD cases were of men (8, 14). Our study showed similar findings of male-predominant CAD with no significant difference between sCAD and mtCAD patients. It is speculated that genetic factors, hormones, and the difference in neck muscles strength and cervical spine stability may play a critical role (14). In contrast, vascular risk factors, such as hypertension, diabetes, hypercholesterolemia, smoking, and alcohol consumption, did not differ between the two groups of sCAD and mtCAD patients, consistent with the CADSIP study (8).

The typical clinical features of CAD patients include craniocervical pain, stroke symptoms, and Horner signs (15). A small group of CAD patients presents only with craniocervical pain without stroke symptoms (15). Craniocervical pain has been reported to be the commonest clinical feature associated with up to 57.8% of patients with mtCAD (10). Our study reported 28.57% of mtCAD patients with clinical features of craniocervical pain, less than previously reported, however, significantly more common than that of patients with sCAD (10). Craniocervical pain may be more likely to be the precipitating or prewarning symptoms for triggering a valuable and urgent presentation (10). Horner signs is another typical clinical feature of CAD with recent research reporting Horner signs as complications in 38.5% of internal carotid arterial dissections and 13.4% of vertebral artery dissection in a pool of 765 patients with CAD (16). Horner signs have been reported to be associated with a more favorable prognosis (16). In our study, there were 13 patients complicated with Horner signs, which was less prevalent compared to what was reported in the literature. Overall, there was no significant difference between sCAD and mtCAD patients in terms of clinical features of Horner signs and stroke symptoms.

Our study reported 28 patients with mtCAD, accounting for 18.9% of all patients with CAD in line with previous studies showing 12–36.5% of patients with CAD associated with mtCAD (8, 17). Recent study from the CADISP group analyzed 966 patients with CAD and found 35.6% of patients with CAD had minor trauma prior to the event, while significantly less association with minor trauma in the control group patients (10). As most of the minor trauma appeared to have minimal impact on daily life, the CADISP group defined “minor trauma” with a more clinically appropriate term “mechanical trigger events.” Cervical spine manipulation, such as gentle neck massage, is usually classified as mechanical trigger events and was reported to precipitate or trigger CADs in up to 30% of the cases (10). However, there is longstanding controversy regarding the association between manipulation and neurovascular complications, mainly because it is clinically impossible to ascertain that we can attribute the minor-trauma-equivalent manipulation to the rare adverse event of CAD if the patients with CAD initially present with head and neck pain for manipulation treatment (18). Our study identified 11 mtCAD patients with a history of cervical spine manipulation, accounting for 39% of the total number of patients with mtCAD. A study of 983 CAD patients suggested that cervical spine manipulation was associated with increased risks of multiple artery dissection (19). Another recent study found that traumatic CAD was more likely to be associated with multiple artery dissection compared to sCAD, (9) consistent with our study results. The mechanism underlying this multiple artery dissection has been suggested in recent study (19) to be precipitated by mechanical traction or twisted force on craniocervical and cerebral arteries. In comparison with sCAD, our study identified that mtCAD was more likely in extra-cranial locations being consistent with another recent study, (20) owing to the greater flexibility of extra-cranial arteries. Furthermore, other studies pointed out that mtCAD in intracranial locations often occurred in the flexible part of the internal carotid and vertebral arteries (7). The underlying mechanism of mtCAD occurring more often extra-cranially rather than intra-cranially has been proposed by another study (21) indicating the more likely direct impact on extra-cranial arteries by surrounding bones, ligaments, and contracting muscles. Extra-cranial arteries were demonstrated to be prone to sustain endothelial injuries during neck movements, such as traction, flexion, and extension, through direct or indirect impact from the surrounding soft tissues, (7) while intracranial arteries tend to avoid these impacts owing to being inside the cranial bony structure.

Digital subtraction angiography is well-regarded as the gold standard for the precise diagnosis of CAD, as it can directly display diagnostic features of dissection, such as intimal flaps, string signs, intravascular stenosis, and occlusion (7, 22). However, DSA has its limitation for clinical application due to its invasive nature with potential complications. In contrast, imaging with CTA and MRA are three-dimensional and non-invasive approaches to be used in detecting most CADs, (9) albeit with their limited capability to detect intramural hematoma. Thus, T1-weighted axial MRI scan with the fat-suppression technique has high sensitivity and specificity for the diagnosis of CAD, in combination with MRA, to visualize both the arterial lumen and arterial wall deficits (1). The innovative high-resolution MRI and three-dimensional acquisition of fat-suppressed sequences with black-blood effects have further improved the precision of CAD diagnosis with the ability to differentiate intimal flap, true or false lumen, and intramural haematoma (23). This sequence uses the double inversion-recovery technique to minimize blood signals, thereby, providing a linear image of the arterial wall (23–25). In our study, all CADs were diagnosed using T1-weighted and T2-weighted contrasted MRI in conjunction with MRA, CTA, or DSA. The two most common imaging features were double lumen (31.75% of patients with CAD) and intramural haematoma (26.35% of CAD patients), respectively. Intramural haematoma has been reported by two other studies (8, 26) with a similar incidence as an imaging diagnostic feature among patients with CAD. In our study, string signs and intramural haematoma were however more commonly presented in patients with mtCAD compared to those of patients with sCAD, while the previous report suggested that intimal flap was the more commonly presented feature among traumatic CAD in comparison to that of sCAD (9). The discrepancy between our study and a previous study was due to the different definitions of mtCAD with a majority of the traumatic CAD patients in the previous study (9) sustaining severe trauma. Moreover, there were no statistical significances comparing mtCAD and sCAD patients in terms of other imaging diagnostic features that include double lumen and dissecting aneurysm, being consistent with other studies.

The majority of CAD patients in our study had only a mild-to-moderate stroke, as indicated by relatively low NIHSS scores (Table 3). These results have been consistent with most of the other studies to imply a favorable outcome with the majority of CAD patients (4, 8, 27, 28). In line with the literature, our study showed no difference between mtCAD and sCAD in terms of stroke severity, while another study demonstrated that mtCAD usually sustained fewer stroke symptoms and functional impairment in comparison to those of sCAD patients. Further study in a large cohort is required to investigate this difference.

The limitations of this study include two aspects with the retrospective design based on a single center and lack of control. Firstly, asymptomatic or mtCAD patients could have been under-diagnosed in the present study with a referral. Secondly, our results suggest that most of the CAD patients have a favorable outcome at 12 months and loss of follow-up occurs in 14.1% of patients. Consequently, long-term favorable functional outcomes could be overestimated.

## Conclusion And Future Perspectives

Our results have identified the different demographic, clinical, and imaging features between mtCAD and sCAD in terms of age, neck pain as presentation, multiple arterial dissection, extra-cranial locations, and intramural haematoma. Patients with mtCAD presented at a much younger age with symptoms of neck pain compared to patients with sCAD. In terms of image features, patients with mtCAD predominantly presented at extracranial sites with more prominent features of multiple site dissection, intramural haematoma, and long tapering stenosis. These unique clinical and imaging features will potentially translate into clinical practice guidelines, such as pharmacological intervention of antiplatelet vs. anticoagulation and duration of these treatments and provide advice on whether immediate surgical intervention is needed in the correct therapeutic window to improve the optimal functional outcome, hence reducing both morbidity and mortality. Future studies with a multi-center design should be planned to continue to expand the current CAD cohort nationally in China and internationally in other continents to obtain long-term functional outcomes and rates of recurrent CAD and stroke. Ultimately, the outcome of the study can be incorporated into internationally accepted clinical guidelines in managing CAD.

## Data Availability Statement

The raw data supporting the conclusions of this article will be made available by the authors, without undue reservation.

## Ethics Statement

The studies involving human participants were reviewed and approved by the Human Research Ethics Committee of The First Affiliated Hospital, Sun Yat-Sen University, Guangzhou, China. The patients/participants provided their written informed consent to participate in this study. Written informed consent was obtained from the individual(s) for the publication of any potentially identifiable images or data included in this article.

## Author Contributions

JZ: conceptualization, data curation, formal analysis, funding acquisition, investigation, methodology, project administration, resources, and writing of review and editing. DX: writing of original draft, writing of review and editing, and submitting author. YW: data curation, formal analysis, investigation, methodology, project administration, resources, software, validation, and writing of original draft. JL, SX, HC, XC, YT, KZ, and GZ: data curation, formal analysis, investigation, methodology, project administration, resources, software, and validation. All authors contributed to the article and approved the submitted version.

## Funding

This study was supported by grants from National Key R&D Program of China (2017YFC1307500), the Natural Science Foundation of Guangdong Province of China (2017A030313575), Guangdong Provincial Key Laboratory of Diagnosis and Treatment of Major Neurological Diseases (2020B1212060017), Guangdong Provincial Clinical Research Center for Neurological Diseases (2020B1111170002), the Southern China International Cooperation Base for Early Intervention and Functional Rehabilitation of Neurological Diseases (2015B050501003 and 2020A0505020004), Guangdong Provincial Engineering Center For Major Neurological Disease Treatment, Guangdong Provincial Translational Medicine Innovation Platform for Diagnosis and Treatment of Major Neurological Disease.

## Conflict of Interest

The authors declare that the research was conducted in the absence of any commercial or financial relationships that could be construed as a potential conflict of interest.

## Publisher's Note

All claims expressed in this article are solely those of the authors and do not necessarily represent those of their affiliated organizations, or those of the publisher, the editors and the reviewers. Any product that may be evaluated in this article, or claim that may be made by its manufacturer, is not guaranteed or endorsed by the publisher.

## References

[1] Debette S Leys D. Cervical-artery dissections: predisposing factors, diagnosis and outcome. Lancet Neurol. (2009) 8:668–78. 10.1016/S1474-4422(09)70084-5 19539238

[2] Nagumo K Nakamori A Kojima S. [Spontaneous intracranial internal carotid artery dissection: 6 case reports and a review of 39 cases in the literature]. Rinsho Shinkeigaku. (2003) 43:313–21. 14503348

[3] Fusca MR Harrigan MR. Cerebrovascular dissections–a review part I: spontaneous dissections. Neurosurgery. (2011) 68:242–57. 10.1227/NEU.0b013e3182012323 21150764

[4] Lee VH Brown RD Jr Mandrekar JN Mokri B. Incidence and outcome of cervical artery dissection: a population-based study. Neurology. (2006) 67:1809–12. 10.1212/01.wnl.0000244486.30455.71 17130413

[5] Piacaroni M Georgiardis D Arnold M Gandjour J Keseru B Fahrni G. Seasonal variability in spontaneous cervical artery dissection. J Neurol Neurosurg Psychiatry. (2006) 77:677–7. 10.1136/jnnp.2005.077073 16614034 PMC2117448

[6] Dittrich R Rohsbach D Heidbreder A Heuschmann P Nassenstein I Bachmann R. Mild mechanical traumas are possible risk factors for cervical artery dissection. Cerebrovasc Dis. (2007) 23:275–81. 10.1159/000098327 17192705

[7] Biller J Sacco RL Albuquerque FC Demaerschalk BM Fayad P Long PH. Cervical arterial dissections and association with cervical manipulative therapy: a statement for healthcare professionals from the American heart association/American stroke association. Stroke. (2014) 45:3155–74. 10.1161/STR.0000000000000016 25104849

[8] Debette S Grond-Ginsbach C Bodenant M Kloss M Engelter S Metso T. Cervical artery dissection ischemic stroke patients (CADISP) group: differential features of carotid and vertebral artery dissections: the CADISP study. Neurology. (2011) 77:1174–81. 10.1212/WNL.0b013e31822f03fc 21900632

[9] Sporns PB Niederstadt T Heindel W Raschke MJ Hartensuer R Dittrich R. Imaging of spontaneous and traumatic cervical artery dissection: comparison of typical CT angiographic features. Clin Neuroradiol. (2019) 29:269–75. 10.1007/s00062-018-0666-4 29374294

[10] Engelter ST Grond-Ginsbach C Metso TM Metso AJ Kloss M Debette S. Cervical artery dissection: trauma and other potential mechanical trigger events. Neurology. (2013) 80:1950–7. 10.1212/WNL.0b013e318293e2eb 23635964

[11] O'Donnell MJ Xavier D Liu L Zhang H Chin SL Rao-Melacini P. Risk factors for ischaemic and intracerebral haemorrhagic stroke in 22 countries (the INTERSTROKE study): a case-control study. Lancet. (2010) 376:112–23. 10.1016/S0140-6736(10)60834-3 20561675

[12] Putaala J Metso AJ Metso TM Konkola N Kraemer Y Haapaniemi E. Analysis of 1008 consecutive patients aged 15 to 49 with first-ever ischemic stroke: the Helsinki young stroke registry. Stroke. (2009) 40:1195–203. 10.1161/STROKEAHA.108.529883 19246709

[13] Rolfs A Fazekas F Grittner U Dichgans M Martus P Holzhausen M. Acute cerebrovascular disease in the young: the stroke in young Fabry patients study. Stroke. (2013) 44:340–9. 10.1161/STROKEAHA.112.663708 23306324

[14] Arnold M Kappeler L Georgiadis D Berthet K Keserue B Bousser MG. Gender differences in spontaneous cervical artery dissection. Neurology. (2006) 67:1050–2. 10.1212/01.wnl.0000237341.30854.6a 17000975

[15] Marciniec M Sapko K Kulczynski M Popek-Marciniec S Szczepanska-Szerej A Rejdak K. Non-traumatic cervical artery dissection and ischemic stroke: a narrative review of recent research. Clin Neurol Neurosurg. (2019) 187:105561. 10.1016/j.clineuro.2019.105561 31634685

[16] Lyrer PA Brandt T Metso TM Metso AJ Kloss M Debette S. Clinical import of Horner syndrome in internal carotid and vertebral artery dissection. Neurology. (2014) 82:1653–9. 10.1212/WNL.0000000000000381 24727317

[17] Rubinstein SM Peerdeman SM van Tulder MW Riphagen I Haldeman Set. A systematic review of the risk factors for cervical artery dissection. Stroke. (2005) 36:1575–80. 10.1161/01.STR.0000169919.73219.30 15933263

[18] Cassidy JD Bronfort G Hartvigsen J. Should we abandon cervical spine manipulation for mechanical neck pain? no. BMJ. (2012) 344:3680. 10.1136/bmj.e3680 22677797

[19] Bejot Y Aboa-Eboule C Debette S Pezzini A Tatlisumak T Engelter S. Characteristics and outcomes of patients with multiple cervical artery dissection. Stroke. (2014) 45:37–41. 10.1161/STROKEAHA.113.001654 24326451

[20] Shin DH Hong JM Lee JS Nasim R Sohn SI Kim SJ. Comparison of potential risks between intracranial and extracranial vertebral artery dissections. Eur Neurol. (2014) 71:305–12. 10.1159/000357867 24662973

[21] Choi MH Hong JM Lee JS Shin DH Choi HA Lee K. Preferential location for arterial dissection presenting as golf-related stroke. AJNR Am J Neuroradiol. (2014) 35:323–6. 10.3174/ajnr.A3768 24184518 PMC7965742

[22] Schwartz NE Vertinsky AT Hirsch KG Albers GW. Clinical and radiographic natural history of cervical artery dissections. J Stroke Cerebrovasc Dis. (2009) 18:416–23. 10.1016/j.jstrokecerebrovasdis.2008.11.016 19900642

[23] Han M Rim NJ Lee JS Kim SY Choi JW. Feasibility of high-resolution MR imaging for the diagnosis of intracranial vertebrobasilar artery dissection. Eur Radiol. (2014) 24:3017–24. 10.1007/s00330-014-3296-5 25017728

[24] Edjlali M Roca P Rabrait C Naggara O Oppenheim C. 3D fast spin-echo T1 black-blood imaging for the diagnosis of cervical artery dissection. AJNR Am J Neuroradiol. (2013) 34: E103–6. 10.3174/ajnr.A3261 23064599 PMC7965613

[25] Cuvinciuc V Viallon M Momjian-Mayor I Sztajzel R Pereira VM Lovblad K. 3D fat-saturated T1 SPACE sequence for the diagnosis of cervical artery dissection. Neuroradiology. (2013) 55:595–602. 10.1007/s00234-013-1141-1 23354947

[26] Matsumoto J Ogata T Abe H Higashi T Takano K Inoue T. Do characteristics of dissection differ between the posterior inferior cerebellar artery and the vertebral artery? J Stroke Cerebrovasc Dis. (2014) 23:2857–61. 10.1016/j.jstrokecerebrovasdis.2014.07.013 25280821

[27] Dziewas R Konrad C Dräger B Evers S Besselmann M Ludemann P. Cervical artery dissection–clinical features, risk factors, therapy and outcome in 126 patients. J Neuro. (2003) 250:1179–84. 10.1007/s00415-003-0174-5 14586598

[28] Metso TM Metso AJ Salonen O Haapaniemi E Putaala J Artto V. Adult cervicocerebral artery dissection: a single-center study of 301 Finnish patients. Eur J Neurol. (2009) 16:656–61. 10.1111/j.1468-1331.2009.02535.x 19220449

